# Repair of a Radial Tear of the Meniscus Augmented With a Biocomposite Scaffold

**DOI:** 10.1016/j.eats.2023.08.005

**Published:** 2023-11-27

**Authors:** Audria Wood, Kaitlin Pyrz, Pearce Lane, Eugene Brabston, Thomas Evely, Aaron Casp, Amit Momaya

**Affiliations:** a Department of Orthopaedics, University of Alabama at Birmingham, Birmingham, Alabama, U.S.A.; b Augusta University/University of Georgia Medical Partnership, Athens, Georgia, U.S.A.

## Abstract

Meniscal tears are a common musculoskeletal pathology in the United States, affecting 61 in every 100,000 people. Surgical repair is indicated for certain tear patterns to reduce the risk of joint degeneration, normalize contact forces in the knee, and help restore normal knee kinematics. However, radial meniscus tear repairs fail to completely heal 38% of the time due to tear characteristics, biology, surgical technique, and inadequate rehabilitation. Recent efforts have incorporated biological augmentation to enhance the healing potential of the meniscus. The BioBrace is a biocomposite scaffold designed to mechanically reinforce tissue and biologically enhance healing. The purpose of this article is to describe an all-inside, meniscal radial tear repair augmented with BioBrace.

Meniscal tears are a common musculoskeletal pathology in the United States, with a prevalence of approximately 12% to 14% and an approximate incidence of 61 in every 100,000 people.^1^ Treatment options for meniscal injury encompass nonoperative modalities, partial meniscectomy, meniscal repair, and meniscal transplantation. This depends on a multitude of factors, including tear morphology. Recently, the incidence of meniscal repair has increased in an attempt to maintain knee function and protect against arthritis in the future. One such type of meniscal tear that can be difficult to treat is a complete radial tear. In a systematic review, radial meniscus tear repairs fail to completely heal 38% of the time.^2^ Failure is often due to varying factors such as tear characteristics, biology, surgical technique, and inadequate rehabilitation.

Recent efforts have been made to enhance the healing potential of meniscal repairs. Biologic augmentations, such as platelet-rich plasma (PRP),^3^ fibrin clots,^4^^,^^5^ marrow stimulation,^6^ and mesenchymal stem cells (MSCs),^7^ have shown promising results.^8^ These interventions have revealed the ability to promote fibrocyte proliferation, extracellular matrix deposition, and neomeniscal tissue formation, highlighting their potential for improving meniscal repair outcomes.^8^

The BioBrace (Biorez) is a biocomposite scaffold that, when implanted onto the meniscus, mechanically reinforces the fibrocartilage and may act to accelerate the healing process of the tear. The BioBrace is composed of bioresorbable poly (L-lactide) microfilaments and highly porous type I collagen. Its biomechanical properties enable it to share loads and assist in the biologic healing while gradually resorbing as the tissue remodels.

We describe our method for an all-inside, radial meniscus repair augmented with BioBrace.

## Surgical Technique

A video illustration of an all-inside, radial lateral meniscus repair augmented with BioBrace is shown in Video 1.

**Fig 1:**
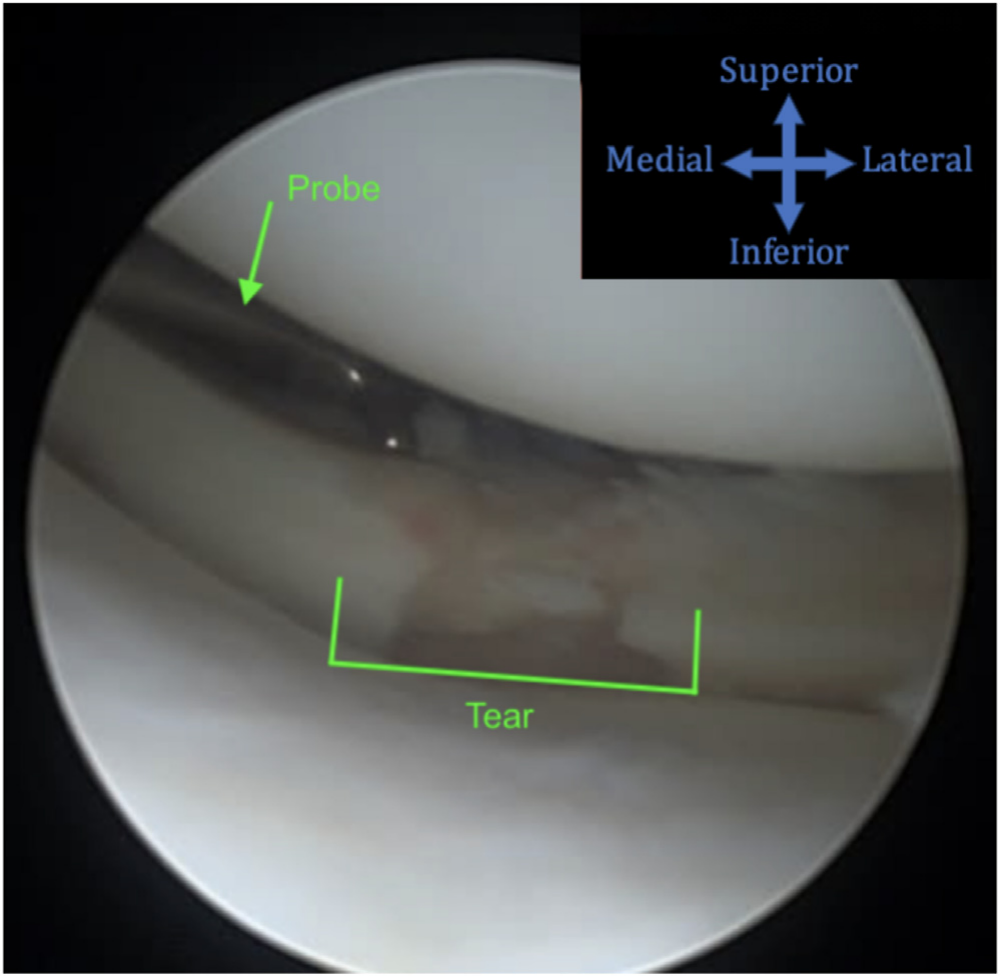
A diagnostic arthroscopy is performed through standard anterolateral and anteromedial portals while the patient is supine. Manual varus force is applied to the knee in a figure-4 position to access the lateral meniscus. A probe then evaluates the lesion before biological preparation is performed. The camera is viewing through the lateral portal while the probe is in the medial portal in a left knee.

Using the standard anterolateral and anteromedial portals, a diagnostic arthroscopy is performed. The knee is placed into a figure-4 position to assess the lateral meniscus. A manual varus force on the knee may help open the lateral compartment further. A radial lateral meniscus tear is shown in Figure 1.

The anterolateral portal is widened to serve as the working portal; the camera is then switched to the anteromedial portal for viewing. The tear is biologically prepared with a shaver, rasp, and spinal needle to optimize healing.

**Fig 2:**
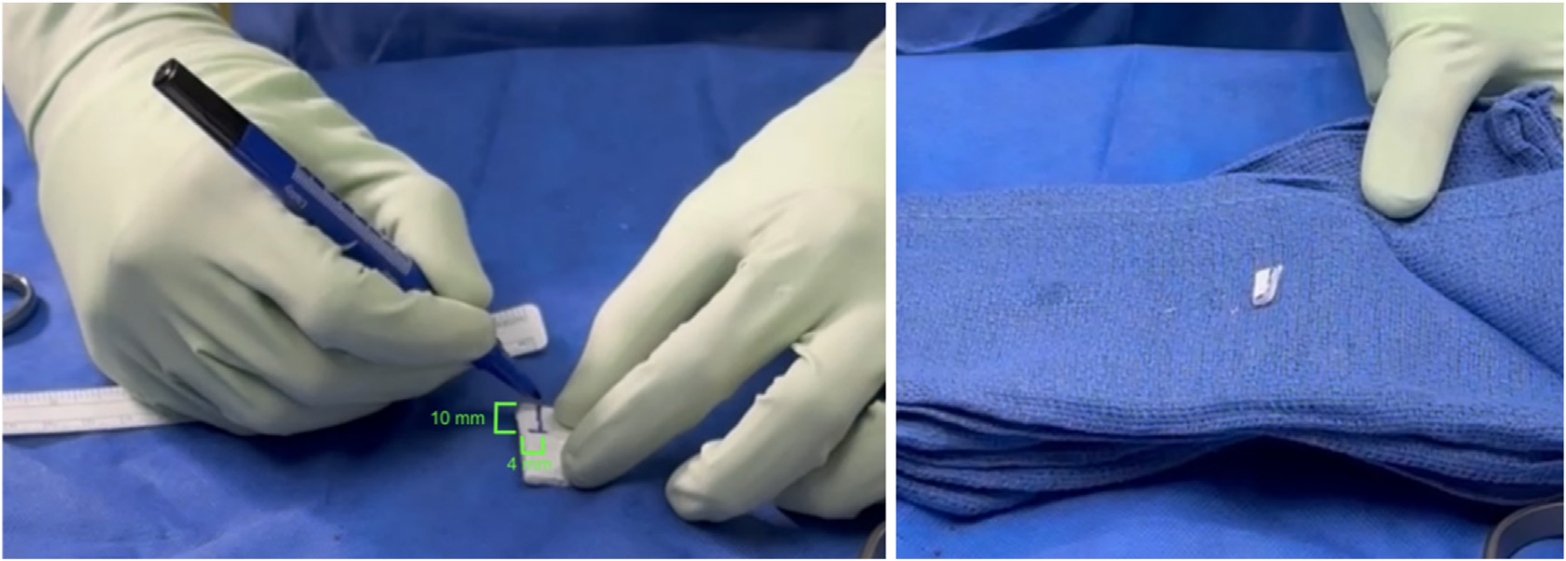
At a separate working station, a 10 × 4-mm area of BioBrace scaffolding is marked and cut before being placed into a saline solution.

On a back table, the BioBrace scaffold is prepared. From the larger 23 × 30-mm BioBrace scaffold, a 10 × 4-mm area is marked and cut (Fig 2). The size of the BioBrace may be adapted based on tear size and pattern. The BioBrace may be saturated with normal saline, PRP, or bone marrow aspirate concentrate solution to hydrate it and make it more pliable.

**Fig 3:**
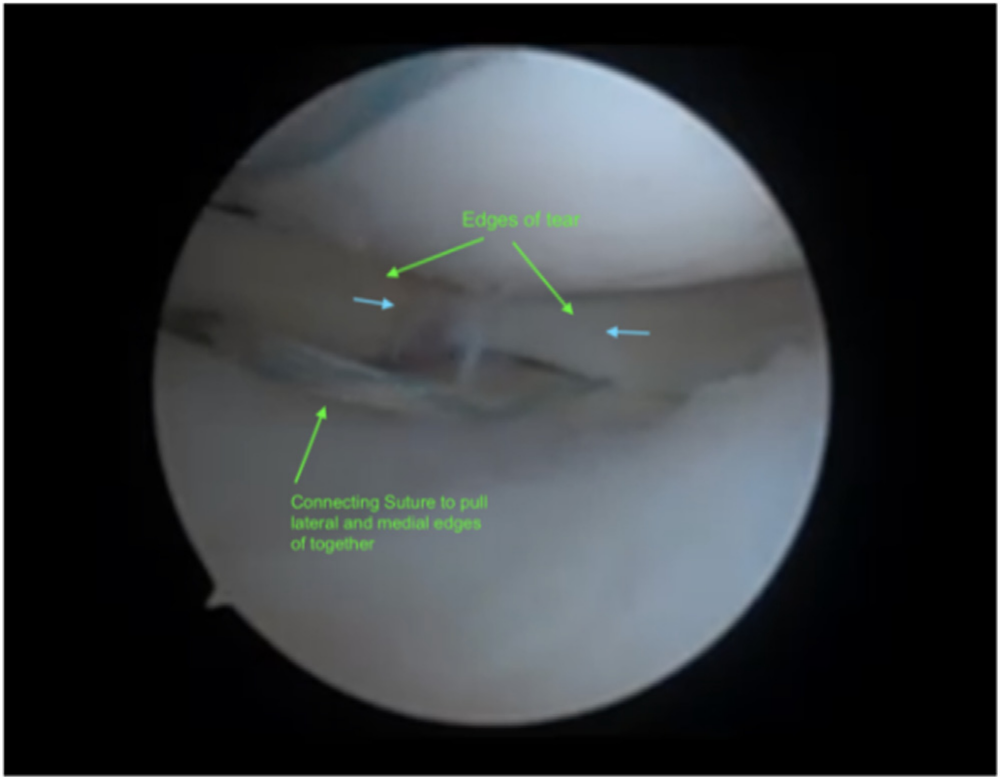
This figure shows the meniscal tear prior to reduction. The patient remains supine with the knee in figure-4 position. A Knee Scorpion Suture Passer (not pictured) passes a 0.9-mm-wide Suture Tape (green arrow) through each of the edges (green arrows) to create a horizontal mattress suture. As the suture is tensioned, the edge should move in accordance with the blue arrows until fully reduced. The suture is neither tied off nor cut at this step. The sutures are through the lateral portal and the camera is through the medial portal in a left knee.

Attention is turned back to the meniscus. A Knee Scorpion Suture Passer (Arthrex) is used to pass 0.9-mm-wide SutureTape (Arthrex) through the medial edge of the tear. In a similar fashion, the opposite end of the suture limb is passed through the lateral edge of the meniscal tear to form a horizontal mattress suture. As the suture is tensioned, the tear is reduced (Fig 3).

**Fig 4:**
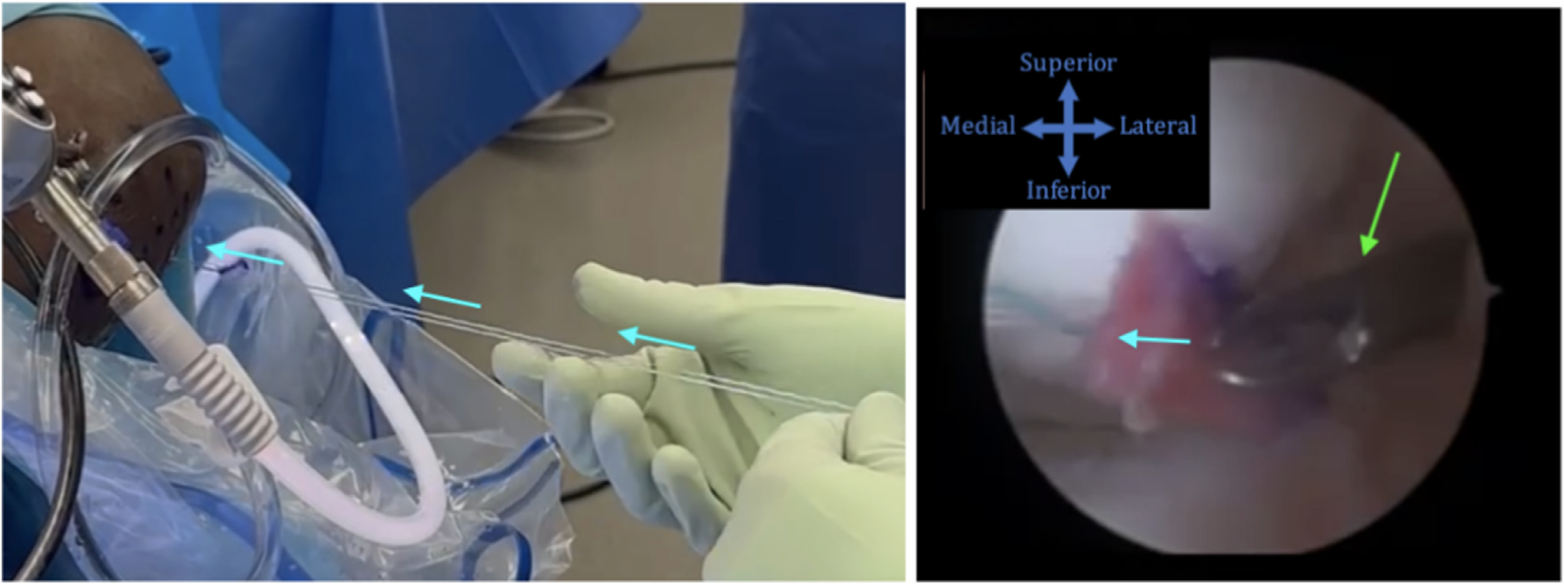
Outside the knee, each suture limb is passed through the respective edge of the BioBrace, using a Keith needle. The patient remains supine with the knee in figure-4 position. The BioBrace is shuttled through the anterolateral portal in the direction of the blue arrows. A knot pusher or ring grasper (green arrow) may be used to continue shuttling the BioBrace onto the meniscal tear site. The sutures may then be tied and cut flush. The camera is in the medial portal and the sutures are tied through the lateral portal of the left knee.

Outside of the knee, each suture limb is passed through an edge of the prepared BioBrace using a Keith needle. The BioBrace is then shuttled into the knee and to the meniscal surface using a knot pusher or a ring grasper (Fig 4). Subsequently, once the BioBrace has been reduced down to the meniscal tear site, the sutures are then tied in standard fashion to reduce the tear and secure the BioBrace across the radial meniscal repair. Once the sutures are tied, the sutures are cut flush.

**Fig 5:**
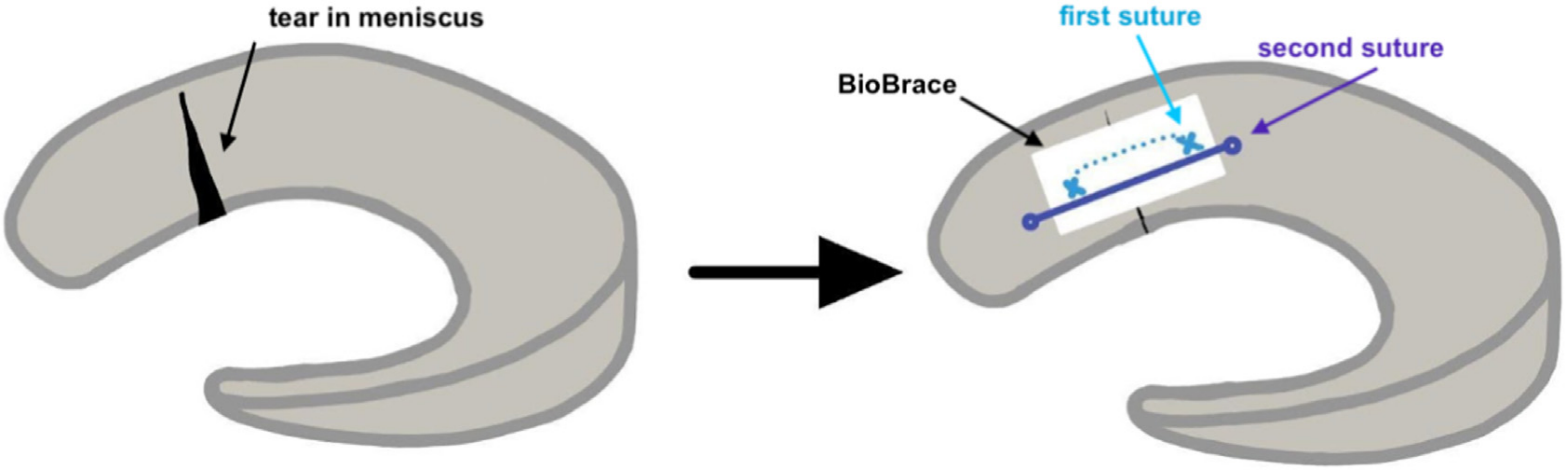
This diagram illustrates BioBrace attachment to the radial tear of the lateral meniscus. The first suture (blue) reduces the tear and attaches the BioBrace. The second suture (purple) further secures the repair construct.

**Fig 6:**
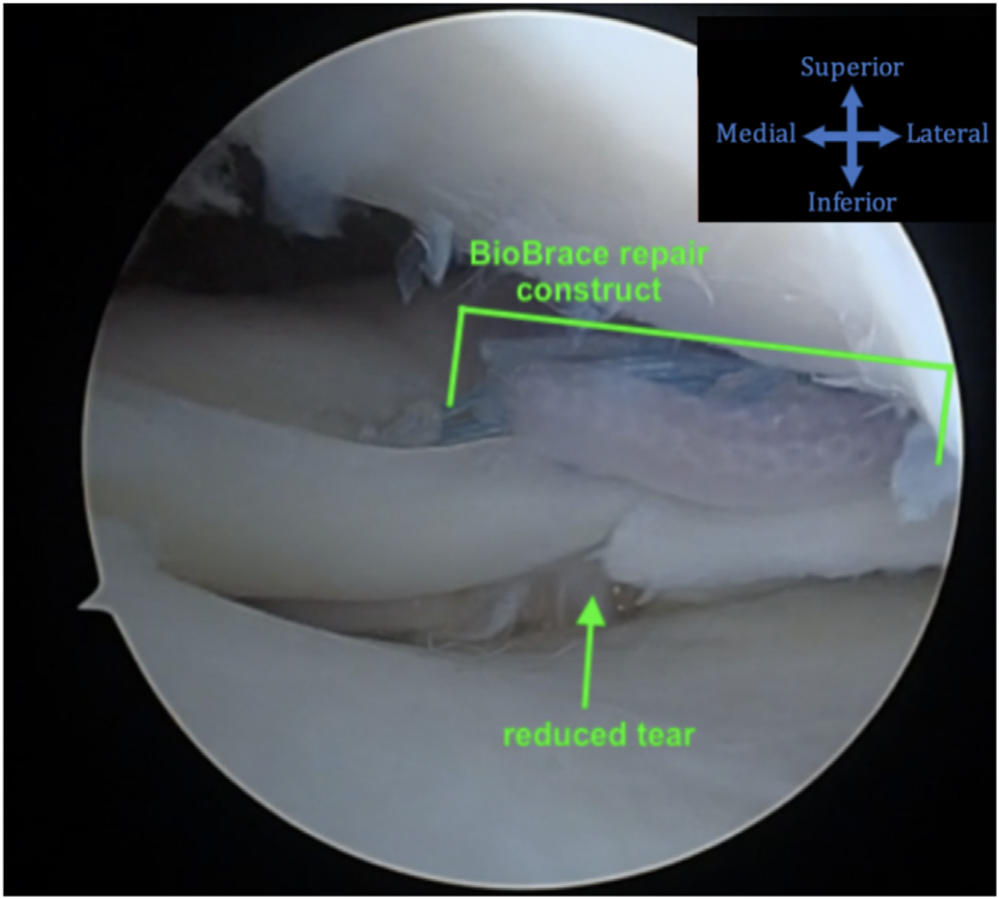
A completed repair of the lateral meniscus radial tear augmented with BioBrace is shown using the anterolateral viewing portal. The patient is supine, and the knee is in figure-4 position. The repair construct is within the green bracket. The reduced lesion is designated by the green arrow. Any remaining pathology may now be addressed.

An all-inside meniscal repair device is then placed in horizontal mattress fashion across the radial meniscal repair. This aids in strengthening the meniscus repair construct and further compressing the BioBrace onto the meniscus repair site. A diagram illustration is located in Figure 5. Repair integrity is then checked with visualization and a meniscal probe (Fig 6). Marrow stimulation may be performed with multiple drill passes or microfracture awl in the notch for further biologic enhancement of the repair. Any concomitant pathology is then addressed as indicated.

Postoperatively, the patient is kept touch-toe weightbearing for approximately 6 weeks. Physical therapy is initiated immediately postoperative, and return to full activity and sports is typically achieved at approximately 4 months after isolated meniscal repair.

## Discussion

This surgical technique describes the use of a biocomposite scaffold implant to augment a meniscus repair. The rationale for biologic augmentation is that it has the potential to improve the biologic integration and vascular regenerative capabilities of the native meniscus while also offering initial mechanical reinforcement at time zero. The application of BioBrace has been implemented in various anatomic regions, such as the medial collateral ligament,^9^ distal biceps tendon,^10^ and rotator cuff,^11^ showing its versatility across different areas of the body.

Recent literature has supported meniscal repair over partial meniscectomy with certain types of tears, especially in younger, active populations. While reoperation rates may be higher in meniscal repair compared to partial meniscectomy, long-term monitoring of meniscal repair cases has revealed superior clinical outcomes and less severe degenerative changes associated with osteoarthritis.^12^

The addition of biologics in sports medicine surgeries has been on the rise, and of particular interest is biologic augmentation of meniscal repairs. This would aim to improve cellular integration, vascularization, matrix deposition, and inflammation reduction at the meniscus repair site.^8^ Notably, the application of PRP,^3^ fibrin clots,^4^^,^^5^ marrow stimulation,^6^ and MSCs^7^ has shown promising results on the basic science level.^13^, ^14^, ^15^ Following repair with biological augmentation, patients have reported significant improvement in functional outcome scores.^8^

However, the extent that these augmented repairs improve outcomes compared to standard repair controls remains uncertain. In a systematic review, Keller et al.^8^ found that only 3 of 10 studies showed significant functional improvement and 1 of 7 studies showed lower revision rates with augmented meniscal repair compared to standard techniques, whereas the remaining studies found no benefit. So while there are some promising results, the role of biologic augmentation to reduce meniscus repair revision surgery rate needs further investigation.^14^^,^^16^

Some biologic enhancements can enhance matrix deposition and tear integration, showing promise for providing mechanical integrity.^15^ Localized delivery of therapeutic factors, scaffold fabrication techniques, decellularized meniscus extracellular matrix, electrospinning of scaffolds, and bioadhesives offer potential solutions for promoting meniscus regeneration and repair, offering an exciting direction for meniscal repairs in the future.^13^ In the meantime, BioBrace offers a combination of biological benefits and enhanced mechanical strength.

In conclusion, the use of BioBrace augmentation for meniscus repairs shows promise in enhancing the biologic healing capabilities of the meniscus while providing initial mechanical reinforcement. This article proposes a reproducible technique for implantation of the BioBrace scaffold for a radial meniscus tear, aiming to improve meniscus repair outcomes. This technique could be adapted for other meniscal tear types. Although further research is needed to fully understand its advantages and limitations compared to standard repair techniques, the potential for improved outcomes and patient satisfaction warrants the continued exploration and investigation. Table 1 and Table 2 discuss the advantages and disadvantages and the pearls and pitfalls, respectively.

**Table 1:** Advantages and Disadvantages

Advantages	Disadvantages
Reproducible technique	Increased cost
Applicable to different types of meniscal repair	Increased time for meniscus repair
Increased biomechanical strength at time zero	Risk of inflammatory response to poly (L-lactide) microfilaments within BioBrace
Enhancement of native meniscus biology with maturation, collagen thickness, and vascularization	Possibility of increased infection risk
	Lack of long-term clinical outcomes
	Requires moderate technical skill

**Table 2:** Pearls and Pitfalls

Pearls	Pitfalls
Ensure appropriate dimensions of BioBrace to match meniscal tear	Care should be directed to make sure the BioBrace lies flat on the meniscus repair site when using the sutures to reduce the tear
Ensure appropriate hydration of the BioBrace in saline, PRP, or BMAC	Avoid suture cut out of BioBrace when threading meniscal suture tape into scaffolding by having a few millimeters of scaffold on either side of the meniscal suture tape

BMAC, bone marrow aspirate concentrate; PRP, platelet-rich plasma.

## Supplementary Data

ICMJE author disclosure forms

Video 1 Here is a step-by-step description for the repair of a radial tear of the meniscus augmented with BioBrace. BioBrace is a biocomposite scaffold for mechanical and biologic enhancement. A standard diagnostic arthoscopy is performed to evaluate the knee, especially the lateral compartments. Once the lesion is identified and prepared, a horizontal mattress suture reduces the lesion and secures BioBrace on the meniscal surface. An additional horizontal mattress suture further secures the repair. The completed repair is evaluated. Any remaining pathology may then be addressed. In this case, there was a full-thickness cartilage defect of the lateral condyle, which was addressed with a mini-open particulated juvenile cartilage transplantation.
